# Serotypes, antimicrobial profiles, and public health significance of *Salmonella* from camels slaughtered in Maiduguri central abattoir, Nigeria

**DOI:** 10.14202/vetworld.2015.1068-1072

**Published:** 2015-09-15

**Authors:** Ibrahim A. Raufu, Ismail A. Odetokun, Fatai S. Oladunni, Mohammed Adam, Ubaidat T. Kolapo, Ganiu J. Akorede, Ibraheem M. Ghali, James A. Ameh, Abdulganiyu Ambali

**Affiliations:** 1 Department of Veterinary Microbiology, Faculty of Veterinary Medicine, University of Ilorin, Ilorin, Nigeria; 2 Department of Veterinary Public Health and Preventive Medicine, University of Ilorin, Nigeria; 3 Department of Veterinary Pathology, University of Ilorin, Nigeria; 4 Department of Veterinary Parasitology and Entomology, University of Ilorin, Nigeria; 5 Department of Veterinary Pharmacology and Toxicology, University of Ilorin, Nigeria; 6 Department of Veterinary Microbiology and Parasitology, University of Abuja, Nigeria; 7 Department of Veterinary Medicine, University of Ilorin, Nigeria

**Keywords:** abattoir, antimicrobial profiles, camel, Nigeria, *Salmonella* serotypes, public health

## Abstract

**Aim:**

This study aimed at determining the serotypes, antimicrobial profiles, and public health importance of *Salmonella* strains from camels slaughtered at Maiduguri central abattoir, Nigeria.

**Materials and Methods:**

Two hundred samples were obtained from camel comprising of intestines, feces, liver, and spleen (n=50 each). Non-lactose fermenting dark center *Salmonella* colonies were identified using standard biochemical techniques, serotyped and subjected to antimicrobial susceptibility test using minimum inhibition concentration method.

**Results:**

Out of the 200 samples collected, 17 were *Salmonella* positive (spleen=7, intestine=6, feces=3, and liver=1) with a prevalence of 8.5%. Five serotypes comprising *Salmonella* Eko, 7 (3.5%), *Salmonella* Uganda, 4 (2.0%), *Salmonella* Amager, 2 (1.0%), *Salmonella* Westhampton, 2 (1.0%), and *Salmonella* Give, 2 (1.0%) were incriminated. Majority of the serotypes were sensitive to the antimicrobials, but one *Salmonella* Amager exhibited resistance to streptomycin, and one each of *Salmonella* Uganda and *Salmonella* Eko were resistant to sulfamethoxazole.

**Conclusion:**

This study revealed the prevalence and the antibiotic resistance profile of newly emerging *Salmonella* from camels in the northeast of Nigeria, which can serve as a means for the transmission of *Salmonella* to human. Therefore, there is a need for the establishment of national *Salmonella* surveillance and control programs.

## Introduction

Camel is a potential source of meat protein in sub-Sahara Africa, the meat is consumed to meet the nutritional requirements in the form of animal protein. Over 2,600 *Salmonella* species are associated with different hosts and virulence [1]. Members of *Salmonella enterica* subspecies enterica are widely distributed in the environment and the gastrointestinal tracts of animals.

In most developed countries, the major causes of human salmonellosis are *Salmonella* Typhimurium and *Salmonella* Enteritidis. However, other serotypes are common in some geographical regions in association with human salmonellosis [2-4]. Infections due to *Salmonella* serotypes are regarded as public health problems worldwide. *Salmonella* prevalence differs between animal species, and the epidemiology of salmonellosis is quite complex due to myriads routes of infection transmission, such routes include transmission from human to animal, from feed to animal, and from animal to animal. Despite the large number of *Salmonella* serotypes, most infections are caused by a minute number of newly emerging serotypes [5].

Aside from *Salmonella* being a major cause of severe illnesses, the emergence of multidrug-resistant (MDR) *Salmonella* strains remains a serious threat to public health; the emergence of resistance to antimicrobials by *Salmonella* from food animals can seriously compromise the treatment of these infections [6]. More importantly, they have been implicated as a major donor of resistance genes to other pathogens or commensals in the gastrointestinal tract [7]. The development of MDR bacteria is usually enhanced by the administration of antimicrobials in food animals.

Third generation cephalosporins and fluoroquinolones are preferred drugs of choice in the management of human invasive salmonellosis; though, fluoroquinolones are also prescribed and administered for many indications in veterinary practices [8].

Salmonellosis has been reported in camels with enteritis [9], nevertheless apparently healthy camels could be carriers of *Salmonella* species [10]. The progressive increase in multi-antimicrobial resistant *Salmonella* strains from human has been associated with indiscriminate administration of antimicrobial agents in food animal production, there are also reports of the spread of antimicrobial resistance through the food chain resulting in a major public health issue [11-13]. In developing countries like Nigeria, *Salmonella* and other zoonotic bacterial pathogens are not routinely cultured, and resistance to commonly employed antimicrobials in public health and veterinary practices is rarely determined.

This study was aimed at determining the serotypes, the antimicrobial resistance patterns and public health importance of *Salmonella* from camels slaughtered for human consumption at Maiduguri central abattoir in the northeastern Nigeria.

## Materials and Methods

### Ethical approval

This study does not require ethical approval; samples were collected from slaughtered camels at Government approved slaughter houses.

### Study location

Maiduguri is located in the arid zone of Borno State covering an area of about 69,436 km^2^ and is situated within latitude 11° 50’ 46” N and longitude 13° 08’ 29” E. It is in the sub-Saharan region with low records of rainfall. The area is in the tropical continental north associated with dry period of 4-8 months (October to May), followed by a short rainy season (late June to early October). The state is located in the North Eastern corner of Nigeria and shares boundaries with Chad to the North East, Cameroon to the East and Adamawa State to the South West. According to the 2005 census, the population is estimated to be 4,558,668 and ranked 12th in the country. Agriculture and livestock husbandry forms the mainstay of the state economy.

### Collection of samples and bacterial isolation

Two hundred samples were randomly collected with 17 samples on average (comprising of spleen, feces, intestine and liver (n=50 each) per sampling trip from different camels slaughtered at three different abattoirs located in Maiduguri Metropolitan (comprising central abattoir, Monday, and Gamboru markets), on a weekly basis from April to September, 2010.

Twenty-five gram each of spleen, feces (direct from the rectum), intestine and liver were aseptically collected from apparently healthy slaughtered camels (male with age ranging between 3 and 6 years) with a universal sample bottles containing 225 ml of nutrient broth (Laboratarios Britania, Buenos Aires, Argentina) and transported in ice box to the veterinary microbiology laboratory of the Faculty of Veterinary Medicine, University of Maiduguri. Samples were incubated at 37°C overnight for 18-24 h. 1 ml of the non-selective pre-enriched culture was inoculated into 9 ml selective broth of Selenite - F (Laboratarios Britania, Buenos Aires, Argentina) and incubated overnight at 37°C for 24 h.

After enrichment, a loop full of the culture was streaked on Deoxycholate Citrate Agar (Park Scientific, Northampton, UK), incubated at 37°C for 24 h. Discrete non-lactose fermenting dark center colonies were picked and streaked (subcultured) onto xylose lysine deoxycholate (XLD) agar (Oxoid Ltd., Hampshire, UK) and incubated for 18-24 h at 37°C.

Presumptive *Salmonella* isolates (presumptive) showing pink/red color with dark center on XLD were picked and confirmed by biochemical characterization in accordance with the standard techniques [14]. The presumptive *Salmonella* isolates were subsequently streaked on nutrient agar (Fluka Biochemika, Steinheim, Germany) slants and incubated at 37°C for 24 h and stored in the refrigerator at 4°C for serotyping and antimicrobial susceptibility tests.

### Serotyping

Presumptive *Salmonella* isolates were shipped to World Health Organization (WHO) National and *Shigella* Center, Bangkok, Thailand for serotyping, this was performed by slide agglutination method, somatic (O) and flagella (H) antigens were characterized by agglutination using hyper immune sera (S and A Reagents Laboratory, Ltd., Bangkok, Thailand) and serotypes were classified based on the Kauffmann–White scheme [15].

### Antimicrobial susceptibility test

Minimum inhibition concentration (MIC) determinations were carried out on all the isolates at Denmark Technical University (DTU-Food), Denmark [16], using a commercial dehydrated panel (TREK Diagnostic Systems Ltd., East Grinstead, England) as summarized below.

### Standardization of inoculum

From a pure culture on a blood agar plate 3-4 colonies were picked and suspended in 4 ml saline in tubes and thoroughly mixed, this was adjusted to McFarland standard 0.5, this was calibrated before use and gently turned with the suspensions upside-down before measuring. The turbidity of inoculum was adjusted to match the standard. The McFarland 0.5 suspension was diluted by mixing 50 µl McFarland 0.5 into 10 ml broth, the suspension was subsequently used for inoculation within 15 min to prevent further growth.

### Inoculation and Incubation

The microtitre plates containing the antimicrobials were inoculated with 50 µl of the inoculum suspension with the aid a Sensititre automatic inoculator (TREK Diagnostic Systems Ltd., East Grinstead, England). Plates were then sealed with the adhesive and incubated at 37°C for 18-22 h. For purity control, 10 µl of the inoculated suspension was uniformly spread on a nutrient agar plate and incubated at 37°C overnight, quality control strain was ran alongside the test strains.

### Reading of MIC

The purity of the inoculum suspension was checked and confirmed to be pure before reporting the results. The plates were read by using the recommended record sheet for the plates, the growth in the 3 positive control wells were checked, the MIC was read and interpreted as the lowest concentration with no visible growth, caution was taken to read the sulphonamides and trimethoprim, with regards to these two antimicrobials the MIC was recorded as the lowest concentration where a reduction of growth by 80-90% can be observed.

Antimicrobials and resistance cut-off values/break point used in the study are as follows: Spectinomycin, (SPE) (R>64 mg/mL); streptomycin, (STR) (R>16 mg/mL); sulfamethoxazole, (SMX) (R>256 mg/mL); tetracycline, (TET) (R>8 mg/mL); and trimethoprim, (TMP) (R>2 mg/mL); ceftiofur, (XNL) (R>2 mg/mL); chloramphenicol, (CHL) (R>16 mg/mL); ciprofloxacin, (CIP) (R>0.06 mg/mL); colistin (COL) (R>8 mg/mL); florfenicol, (FFN) (R>2 mg/mL); gentamicin, (GEN) (R>2 mg/mL); nalidixic acid, (NAL) (R>16 mg/mL); neomycin, (NEO) (R>8 mg/mL); ampicillin, (AMP) (R>4 mg/mL); amoxicillin-clavulanic acid, (AUG) (R>4 mg/mL); apramycin, (APR) (R>4 mg/mL).

Epidemiological cut-off values were interpreted in accordance to current European Committee on Antimicrobial Susceptibility Testing harmonized breakpoints (www.eucast.org) with the exceptions to interpretation of APR, AUG, COL, FFN, SPE, and XNL where Clinical and Laboratory Standards Institute [17] and clinical breakpoints were used, NEO and STR were interpreted according to research conducted at DTU-Food.

## Results

From the 200 samples analyzed, 17 were positive for various species of *Salmonella* representing a prevalence of 8.5%. Five serotypes comprising *Salmonella* Eko, *Salmonella* Uganda, *Salmonella* Amager, *Salmonella* Westhampton, and *Salmonella* Give were identified as shown in Table-1. On the basis of strain prevalence, *Salmonella* Eko constitutes 7 (41.2%), *Salmonella* Uganda, 4 (23.5%), *Salmonella* Amager, 2 (11.8%), *Salmonella* Westhampton, 2 (11.8%), and *Salmonella* Give, 2 (11.8%) while the prevalence of serotypes on the basis of the sample types showed spleen and intestine presenting a higher incidence of serotypes, with 7 (14.0%) and 6 (12.0%) respectively, this is followed by feces 3 (6.0%) and liver 1 (2.0%) as presented in Table-1.

**Table-1:** Serotype-sample prevalence of *Salmonella* isolated from camel in Maiduguri, sub-Sahara Nigeria.

Serotypes/samples	Specific serotype identified	No of serotype obtained	Prevalence in %
Serotypes	*Salmonella* Eko	7	41.2
	*Salmonella* Uganda	4	23.5
	*Salmonella* Amager	2	11.8
	*Salmonella* Give	2	11.8
	*Salmonella*	2	11.8
	*Westhampton*		
Total		17	100.0
Samples	Origin		
	Spleen	7	14.0
	Intestine	6	12.0
	Faeces	3	6.0
	Liver	1	2.0
Total		17	100.0

Most of the serotypes are sensitive to all the antibiotics with the exception of one *Salmonella* Amager isolate from spleen that exhibited resistance to streptomycin, and one each of *Salmonella* Uganda and *Salmonella* Eko isolate from spleen and faeces respectively that exhibited resistance to sulfamethoxazole as shown in Table-2.

**Table-2:** Antimicrobial resistance profiles among *Salmonella* serotypes isolated from camel in Maiduguri, subSahara Nigeria.

*Salmonella* Serotype	Number of isolate	Number (%) of isolates resistant to various antimicrobial agents at the indicated breakpoints (μg/mL)^a^																	

		AUG	AMP	APR	FOT	CHL	CIP		COL	FFN	GEN	NAL	NEO	SPE	STR	SMX	TET	TMP	XNL
																
		≥32	>8	>32	>0.5	>16	0,0641	>1	>2	>16	>2	>16	>4	>64	>16	>256	>8	>2	>2
Eko	7	0	0	0	0	0	0	0	0	0	0	0	0	0	0	1 (14)	0	0	0
Uganda	4	0	0	0	0	0	0	0	0	0	0	0	0	0	0	1 (25)	0	0	0
Amager	2	0	0	0	0	0	0	0	0	0	0	0	0	0	1 (50)	0	0	0	0
Westhampton	2	0	0	0	0	0	0	0	0	0	0	0	0	0	0	0	0	0	0
Give	2	0	0	0	0	0	0	0	0	0	0	0	0	0	0	0	0	0	0
Total	17	0	0	0	0	0	0	0	0	0	0	0	0	0	1	2	0	0	0

Abbreviations of the antimicrobials were AMP=Ampicillin, AUG=Amoxicillin+clavulanic acid, APR=Apramycin, FOT=Cefotaxime, XNT=Ceftiofur, CHL=Chloramphenicol, CIP=ciprofloxacin, COL=Colistin, FFN=Florfenicol, GEN=Gentamicin, NAL=Nalidixic acid, NEO=Neomycin, SPE=Spectinomycin, STR=Streptomycin, SMX=Sulphamethoxazole, TET=Tetracycline, TMP=Trimethoprim

## Discussion

This study report the isolation of *Salmonella* species from camel, this is the first report of these serotypes in camel from Maiduguri, sub-Sahara Africa. The study highlighted the possibility and potential risk of acquiring salmonellosis from consumption of camel meat slaughtered at the Maiduguri central abattoir, north eastern Nigeria. It also revealed the presence of *Salmonella* serotypes in a significant proportion (8.5%) of camel in the north east of Nigeria. This outcome corroborated a previous work by Wernery and Kaaden [18], which classified camels as a reservoir for *Salmonella* infection; hence, camel meat could be a potential hazard for public health. Camels and its products could be a potential reservoir for salmonellosis not only for the camels but also to human and other animal species. *Salmonella* Eko, *Salmonella* Uganda, *Salmonella* Amager, *Salmonella* Westhampton, and *Salmonella* Give can therefore be classified as the major serotypes causing camel salmonellosis in Nigeria.

*Salmonella* serotypes obtained from this study are uncommon serotypes in Nigeria and have not been reported in camels as evidenced from several studies in the past. In United Arab Emirate, *Salmonella* Frintrop, and *Salmonella* Hind marsh as the most commonly incriminated and host adapted *Salmonella* serotypes from camel [19,20]. Similarly, study carried out between 1987 and 1991 by Wernery [21] implicated *Salmonella* Saintpaul, *Salmonella* Frintrop, and *Salmonella* Hindmarsh as the most frequent of *Salmonella* serotypes recovered from camel in UAE. Study carried out in Egypt by Mohammed and Suelam [22] reported *Salmonella* enteritidis and *Salmonella* typhimurium as the prevalent serotypes which are in contrast to the outcome of this study.

The overall *Salmonella* prevalence reported in various studies were at variance to the outcome of this study, though the disparities vary from one study to another, prevalence of 4.7% in UAE [21], 5.6% in Egypt [22] and 7.4% in UAE [19] these are lower when compared to 8.5% obtained from this study. Similar study in Ethiopia [10] reported an overall prevalence of 16.2%.

This study reported a prevalence of fecal *Salmonella* to be 6%, while 15.1% prevalence was reported in Ethiopia [10] 13.9% in Egypt [22], and 4.3% in UAE [21].

Molla *et al*. [10] reported a prevalence of 11.8% from liver as against the 2% reported in this study and 0% reported by Mohammed and Suelam in Egypt. Previous studies on camel spleen reported a prevalence of 14.3% in Ethiopia [10] which is comparable to 14% from this study.

Camels like other animals can become a chronic carrier of *Salmonella* organisms and subsequently excretes the organisms in their feces intermittently, especially at times of stress such as during parturition, concurrent diseases, starvation, overcrowding and transport [22]. The carrier animals constitute a threat to camels and other species of animals; it can also become a health hazard to human through contact with contaminated carcasses or by ingestion of other contaminated edible products.

MDR *Salmonella* are of worldwide interest because they reduce the therapy options in human and veterinary medicine [6]. This study showed that MDR *Salmonella* were not incriminated in camel which indicates that the management system employed does not promote the indiscriminate use of antimicrobials or are being used as prescribed by the veterinary officer in charge. Furthermore, the susceptibility to important antimicrobials used for human/animals like fluoroquinolones and cephalosporins, is a positive development and this implies a need for the establishment of a coordinated surveillance, control and monitoring, program for *Salmonella* in the study area to prevent the establishment of resistance to these antimicrobials by the newly emerging serotypes.

## Conclusion

The output of this study provides vital information on the camel as asymptomatic carriers of *Salmonella* in the study region, it equally revealed the trends in the prevalence of newly emerging or rarely reported *Salmonella* serotypes, and the antimicrobial resistance profile of *Salmonella* from camel in north east of Nigeria, this study can be adopted as a base for *Salmonella* control, prevention and therapeutic strategies.

## Authors’ Contributions

IAR, JAA, AA designed and planned this research work. IAR, GJA, IAO, FSO, IMG, UTK, MA collected samples, and performed laboratory investigation. IAR, IAO, JAA and AA drafted and revised the manuscript. All authors read and approved the final manuscript.
